# An image dataset for surveillance of personal protective equipment adherence in healthcare

**DOI:** 10.1038/s41597-024-04355-0

**Published:** 2025-01-17

**Authors:** Wanzhao Yang, Mary S. Kim, Genevieve J. Sippel, Aaron H. Mun, Kathleen H. McCarthy, Beomseok Park, Aleksandra Sarcevic, Marius George Linguraru, Ivan Marsic, Randall S. Burd

**Affiliations:** 1 Department of Electrical and Computer Engineering, Rutgers University, Piscataway, NJ 08854 USA; 2 Division of Trauma and Burn Surgery, Children’s National Hospital, Washington, DC 20010 USA; 3 College of Computing and Informatics, Drexel University, Philadelphia, PA 19104 USA; 4 Sheikh Zayed Institute for Pediatric Surgical Innovation, Children’s National Hospital, Washington, DC 20012 USA; 5 Departments of Radiology and Pediatrics, George Washington University School of Medicine and Health Sciences, Washington, DC 20037 USA

**Keywords:** Lifestyle modification, Research data

## Abstract

Proper personal protective equipment (PPE) use is critical to prevent disease transmission to healthcare providers, especially those treating patients with a high infection risk. To address the challenge of monitoring PPE usage in healthcare, computer vision has been evaluated for tracking adherence. Existing datasets for this purpose, however, lack a diversity of PPE and nonadherence classes, represent single not multiple providers, and do not depict dynamic provider movement during patient care. We introduce the Resuscitation Room Personal Protective Equipment (R2PPE) dataset that bridges this gap by providing a realistic portrayal of diverse PPE use by multiple interacting individuals in a healthcare setting. This dataset contains 26 videos, 10,034 images and 123,751 bounding box annotations for 17 classes of PPE adherence and nonadherence for eyewear, masks, gowns, and gloves, and one additional head class. Evaluations using newly proposed metrics confirm R2PPE exhibits higher annotation density than three established general-purpose and medical PPE datasets. The R2PPE dataset provides a resource for developing computer vision algorithms for monitoring PPE use in healthcare.

## Background & Summary

The US Centers for Disease Control and Prevention (CDC) provides guidance about appropriate personal protective equipment (PPE) to protect healthcare workers and provide infection control (https://archive.cdc.gov/#/details?url=https://www.cdc.gov/coronavirus/2019-ncov/downloads/A_FS_HCP_COVID19_PPE.pdf). Despite these recommendations, PPE adherence among healthcare providers was often low during the COVID-19 pandemic, even in settings at high-risk for infection transmission^1–3^. This lack of PPE adherence partly accounts for the 12-fold increased risk of COVID-19 infection among healthcare workers during the pandemic^4^. Without appropriate PPE, infection risk rises above one percent for every minute of exposure to an infectious person^5^. To address PPE nonadherence, hospitals have implemented PPE auditors to monitor infection control practices^6^. While in-person PPE monitoring has improved PPE adherence and reduced infection risk, human observations are intermittent due to staff constraints, prone to error, and require additional personnel during staffing shortages^6,7^(https://www.theguardian.com/australia-news/2020/aug/04/victorian-nurses-ask-for-urgent-ppe-as-more-than-730-health-workers-sick-with-covid-19). To address the limitation of human observers, previous studies have evaluated computer vision as an approach for monitoring PPE adherence in healthcare settings^8–10^. A computer vision system may enable continuous, scalable monitoring and generate large-scale data for infection control analyses. Annotated video datasets are needed that represent the complexity and diversity of PPE requirements in different medical domains to develop adequate computer vision systems for clinical deployment.

Most public image datasets available for training computer vision algorithms to detect PPE in medical domains focus on a single PPE type, most commonly masks^11,12^(https://humansintheloop.org/resources/datasets/medical-mask-dataset/). To our knowledge, only three datasets include the four essential categories of PPE specified by the CDC: eyewear, masks, gowns, and gloves^8,9,13^. The first dataset contains 1,350 web-sourced images with 6,569 annotations featuring individuals wearing eyewear, masks, gowns, and gloves in diverse settings^8^. While some images obtained from public surveillance cameras may show partial occlusion of PPE caused by motion, other images are staged with an intent to display full PPE visibility. Although this dataset includes examples of mask nonadherence, it provides annotations only for complete adherence with eyewear, gloves, and coveralls. The second dataset, Medical Personal Protective Equipment (CPPE-5), contains 1,029 images with 4,698 annotations. It is also web-sourced and includes examples of eyewear, masks, coveralls, and gloves^13^. Similar to the first dataset, the CPPE-5 dataset does not include examples of PPE nonadherence. While N95 masks and surgical masks are included in the dataset, both are given a single label despite providing different levels of protection. The third dataset, Medical Staff Dress Code Dataset (MSDCD), contains 5,233 images with 28,186 annotations of six PPE categories: headcover, eyewear, masks, coveralls, gloves, and shoe covers^9^. This dataset includes annotation of adherent and nonadherent PPE images but, again, uses a single label for all mask types. The images were also taken against a white background to minimize visual distractions and simplify object detection.

To address the limitations of current PPE datasets in medical domains, we developed the Resuscitation Room Personal Protective Equipment (R2PPE) dataset. This dataset is designed to support the development and evaluation of targeted object detection models for infection control. The R2PPE dataset contains 10,034 images and 123,751 annotations focusing on four PPE categories (masks, gowns, eyewear, and gloves) outlined by the CDC for COVID-19 prevention and control (https://archive.cdc.gov/#/details?url=https://www.cdc.gov/coronavirus/2019-ncov/hcp/non-us-settings/overview/index.html, https://www.cdc.gov/healthcare-associated-infections/media/pdfs/ppe-sequence-p.pdf). This dataset contains more images and annotations than the three published medical PPE datasets. It covers 17 classes of adherence and nonadherence (Table 1), with eight of them having not been represented in any previous medical PPE datasets. Unlike images of current medical PPE datasets, the R2PPE videos were captured in an actual medical setting equipped with a range of medical tools and equipment (e.g., otoscope, stethoscope, and oxygen mask), providing a realistic context for real-world clinical application. The dataset includes images from video generated by simulating patient assessments on a mannequin, replicating crowded environments encountered in some clinical settings, and object occlusions common in real-world images. Individuals were not in a fixed position but were moving to visually represent a diversity of PPE use at various camera angles and distances. Using a density metric, we compared object clustering in R2PPE with other general-purpose and medical PPE datasets. We also report the results of applying three object detection models on R2PPE, providing a baseline for future analyses.

**Table 1:** Definitions and visual indicators for the 17 annotated classes of PPE adherence and nonadherence in the R2PPE dataset.

PPE Item	Adherence	Class	Visual Cues
Mask	Absent	Mask Absent (MA)	The mask is either not worn or worn with the nose and mouth exposed.
	Inadequate	Mask Incomplete (MI)	The mask is pulled down below the nose but covers the mouth.
		Regular Mask Complete (RC)	A regular mask is light purple or yellow with two white ear straps covering both the nose and mouth.
	Complete	N95 Complete (NC)	N95 masks protrude from the face, can be white or blue, and have white or yellow Straps (S) around the back of the head.
Gown	Absent	Gown Absent (GA)	No gown is worn; shoulders, arms, and torso are visible.
	Inadequate	Gown Incomplete (GI)	The gown is worn, but the necktie is not secured, and/or the shoulder is visible.
	Complete	Gown Complete (GC)	The yellow gown covers the provider’s shoulders, arms, and torso with strings tied around the neck.
Eyewear	Absent	Eyewear Absent (EA)	No eyewear is worn.
	Inadequate	Prescription Glasses (PG)	Prescription glasses lack side and top shields.
		Safety Glasses (SG)	Safety glasses have a clear or colored frame with side and top shields.
		Face Shield Incomplete (FI)	The blue or gray foam of the face shield rests atop the head with the shield at an angle to the face.
	Complete	Face Shield Complete (FC)	The face shield rests on the forehead with its clear plastic shield aligned with the face.
		Goggles (GG)	Durable, circumferential eyewear with a snug fit around the top half of the face, secured with a wide elastic band across the back of the head.
		Powered Air Purifying Respirator (PR)	The respirator resembles a helmet.
Gloves	Absent	Glove Absent (HA)	The provider’s hand is visible.
	Complete	Glove Complete (HC)	A blue glove covers the hand.

The non-PPE head class is not included in this table.

## Methods

### Research Site and Medical Simulations for Image Capturing

The dataset images were captured in the emergency department at Children’s National Hospital. The Children’s National Hospital Institutional Review Board approved the study (IRB ID: STUDY00000360). Twelve individuals participated in 19 simulations wearing different PPE combinations. Hospital staff were recruited in person for participation in simulation videos based on their availability and interest in the study. Due to the nature of the dataset, anonymization of individuals was not feasible. Participation in the video recording required signing an informed consent form that addressed the risks of loss of confidentiality from fully visible faces. The consent also included that the dataset could be used in presentations, publications, or public datasets. Participants were informed that their involvement could contribute to computer vision research on PPE usage but that they would derive no personal benefits from its use. All interested staff were allowed to participate with appropriate consent, increasing the diversity of PPE representations in the dataset.

**Fig. 1:**
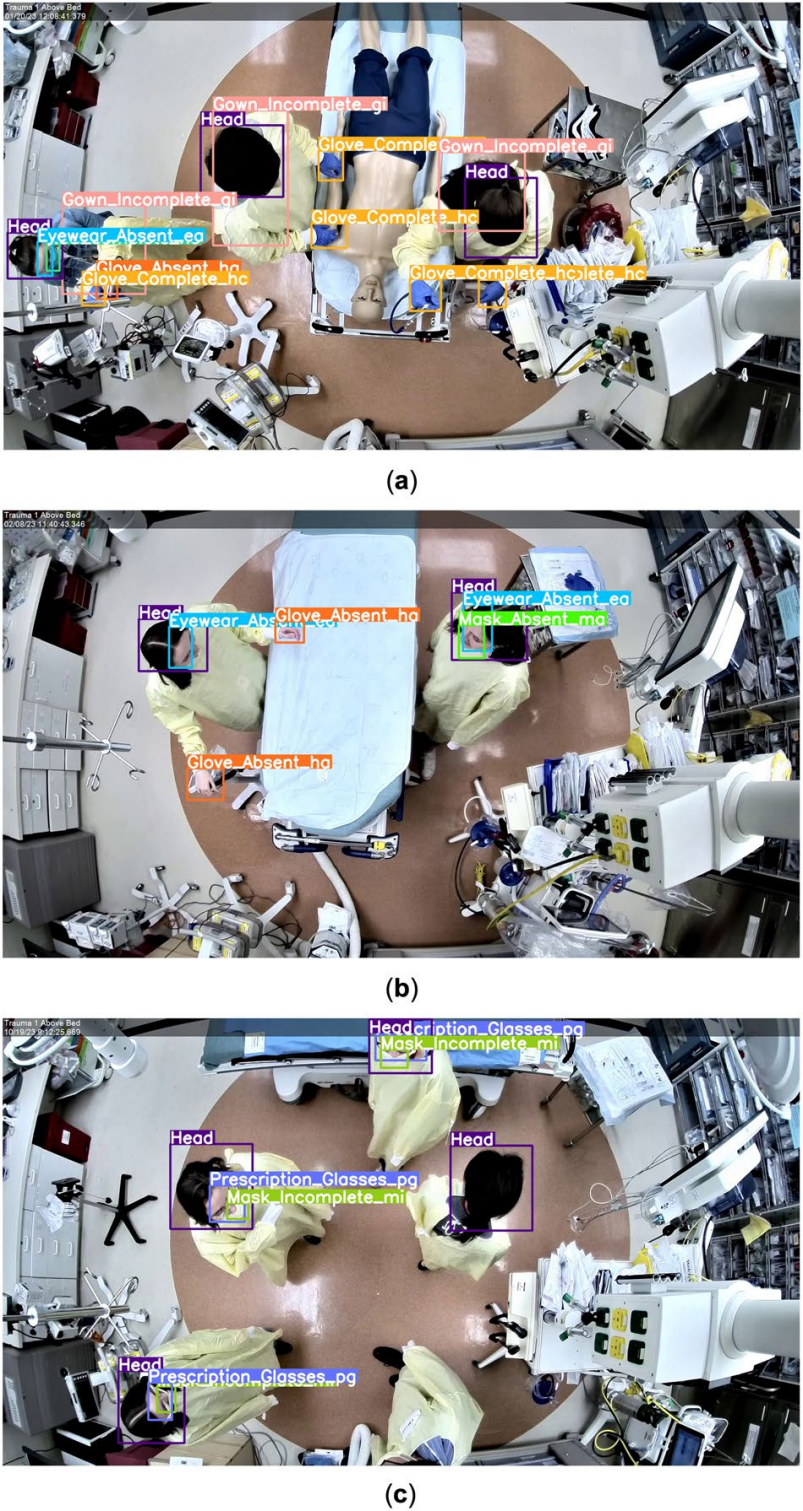
Sample images from R2PPE obtained from different medical setups. Bounding boxes are overlaid on items of interest to indicate the PPE adherence and nonadherence classes in different colors. (**a**) Scenario 1: a mannequin placed on a patient bed. (**b**) Scenario 2: a bed with no mannequin. (**c**) Scenario 3: individuals walking around.

Thirteen individuals participated in 26 simulations wearing different combinations of medical masks, gowns, eyewear and gloves. The dataset includes 17 PPE classes representing three adherence levels (complete, inadequate, and absent) (Table 1) and one additional head class. We defined PPE adherence and nonadherence for each PPE category based on CDC guidelines for airborne precautions (https://www.cdc.gov/infection-control/hcp/isolation-precautions/?CDC_AAref_Val=https://www.cdc.gov/infectioncontrol/guidelines/isolation/index.html, https://archive.cdc.gov/#/details?url=https://www.cdc.gov/niosh/topics/eye/eye-infectious.html). The dataset provides multiple examples for each PPE class based on availability at the institution (e.g., blue and white N95 masks and disposable and reusable gowns). The videos were filmed on 13 separate days to capture variations in attire, which may influence PPE detection. Videos of simulations were recorded using a 30 frames per second camera mounted on the ceiling. We filmed 26 videos based on three simulation scenarios (Figure 1, Table 2). In the first scenario, individuals performed different medical tasks on a mannequin placed on a bed at the center of the room (e.g., application of oxygen supplementation, manual blood pressure measurement, and manual cervical spine stabilization). This setup simulated provider activities and postures seen during an emergency medical assessment that may affect PPE visibility. In the second scenario, individuals walked around and stood next to a patient bed in the center of the room under the camera. This setup provided examples of PPE visibility in an uncrowded, structured medical setting. Individuals in the third scenario walked around an empty room, allowing the camera to capture PPE items at different angles and distances. This setup generated images with a diversity of visual representations of PPE adherence and nonadherence. In all three scenarios, individuals were free to change their PPE and leave and reenter the camera view as in real-world practice. Each video had two to six individuals and an average duration of 427.5 seconds (range 67 to 899 seconds). The variations in the individual count were to simulate both crowded and uncrowded environments. The simulations were designed to approximate real clinical events, as actual clinical videos cannot be shared publicly due to patient and provider confidentiality.

**Table 2:** Simulation Log.

Video Name	Individual Count (n)	Frame Count (n)	PPE Classes
Scenario 1: Clinical simulation of patient care on a mannequin on patient bed			
clinical_sim_1	6	600	ma, mi, rc, nc, s, ga, gi, gc, ea, ha, hc, head
clinical_sim_2	6	540	ma, mi, rc, nc, s, ga, gi, gc, ea, fc, sg, ha, hc, head
clinical_sim_3	6	480	ma, rc, nc, s, ga, gi, gc, ea, fc, sg, ha, hc, head
clinical_sim_4	3	300	ma, mi, rc, nc, s, gi, ea, ha, hc, head
clinical_sim_5	3	195	mi, rc, ga, gi, ea, ha, hc, head
Scenario 2: Walking or standing next to patient bed			
bed_sim_1	4	76	ma, rc, nc, s, ga, gi, gc, ea, fc, sg, ha, hc, head
bed_sim_2	3	113	mi, gc, ea, fi, head
bed_sim_3	3	491	ma, mi, nc, gc, fi, head
bed_sim_donning_doffing	3	225	ma, mi, rc, nc, s, ga, gi, gc, ea, ha, hc, head
bed_sim_ma	2	434	ma, mi, rc, ga, gi, gc, ea, ha, hc, head
bed_sim_n95	4	337	ma, mi, nc, s, ga, gi, gc, ea, fc, fi, ha, hc, head
Scenario 3: Walking around the room			
move_sim_1	3	232	ma, mi, rc, nc, s, ga, gi, gc, ea, ha, hc, head
move_sim_2	3	780	ma, mi, rc, nc, s, ga, gi, gc, ea, ha, hc, head
move_sim_3	4	234	ma, mi, rc, nc, s, ga, gi, gc, ea, fc, sg, ha, hc, head
move_sim_4	4	300	ma, mi, rc, nc, s, ga, gi, gc, ea, fc, ha, hc, head
move_sim_5	4	622	ma, mi, rc, ga, ea, fc, pg, ha, head
move_sim_6	6	68	mi, rc, ga, gi, fi, pg, gg, ha, hc, head
move_sim_7	6	67	mi, rc, ga, gi, ea, fc, pg, gg, ha, hc, head
move_sim_8	3	599	ma, mi, nc, s, ga, gc, sg, pg, ha, head
move_sim_fi	5	391	ma, mi, rc, gi, gc, fc, fi, head
move_sim_gg	5	508	ma, mi, rc, gc, gg, head
move_sim_gg2	3	354	mi, rc, nc, s, ga, gc, ea, sg, gg, ha, hc, head
move_sim_papr	3	369	gc, pr, head
move_sim_papr_gi	3	849	ma, rc, nc, sg, gi, ea, pr, ha, hc, head
move_sim_pg	5	438	mi, rc, sg, pg, ha, head
move_sim_sg	5	432	mi, rc, nc, gc, sg, head, person

Frame count excludes empty frames with no annotations.

### Dataset Annotation

The simulation videos were uploaded to the V7 video annotation tool (https://www.v7labs.com/) at one frame per second. Eight research members annotated the images frame by frame, outlining bounding boxes for each PPE item. We maintained annotation consistency using a data dictionary that provided visual cues for PPE types and adherence classes (Table 1). The bounding boxes were labeled with the PPE type and adherence class. In cases where PPE was absent or inadequate (e.g., mask lowered below the nose), we marked the nonadherence level (e.g., mask incomplete). When the ground truth was ambiguous to the human annotator, we omitted the label. In addition to PPEs, a separate head bounding box was added to aid in the development of algorithms focused on PPE worn on the head.

## Data Records

**Fig. 2:**
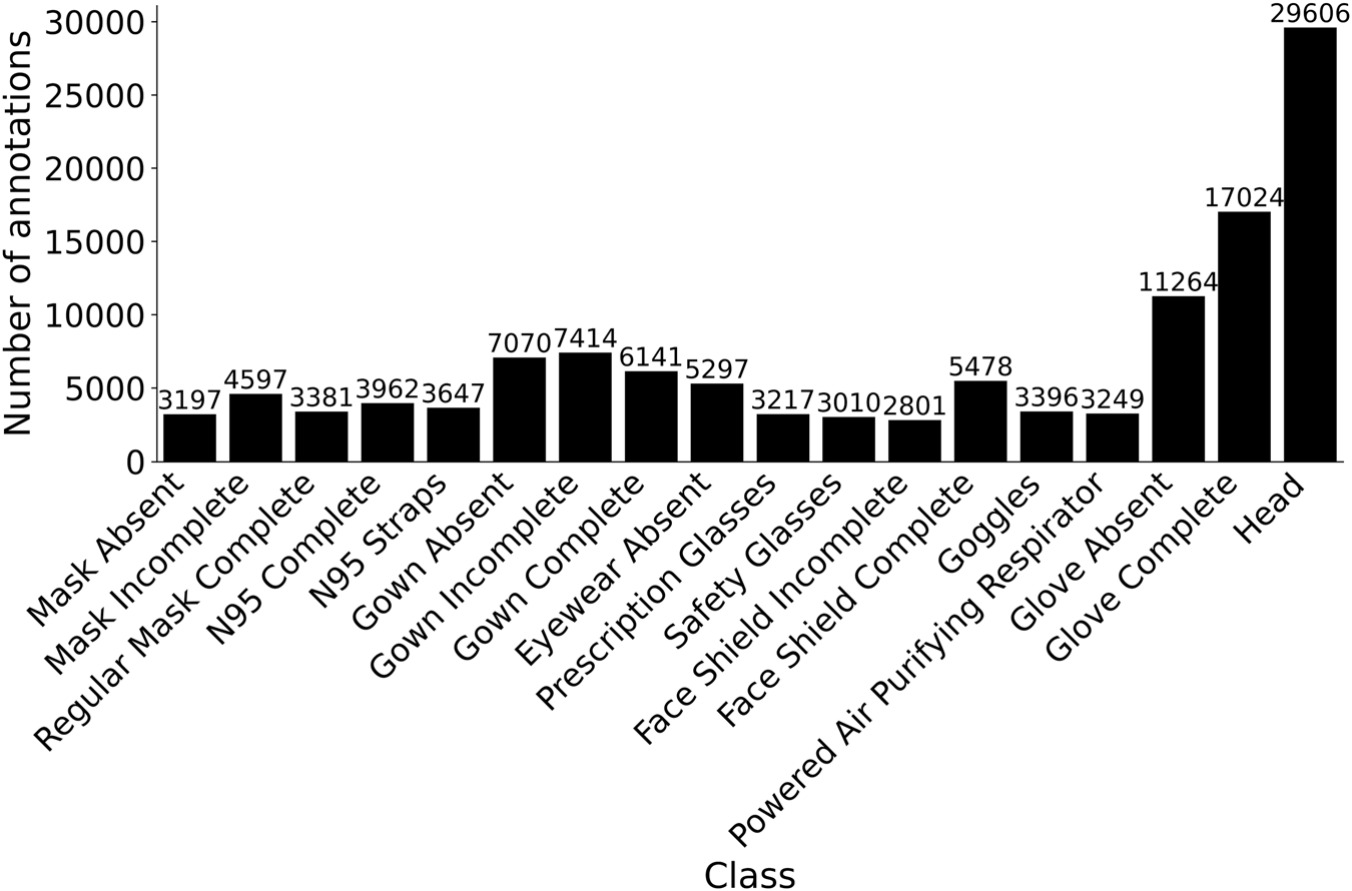
Distribution of PPE adherence and nonadherence classes represented in the dataset images.

**Fig. 3:**
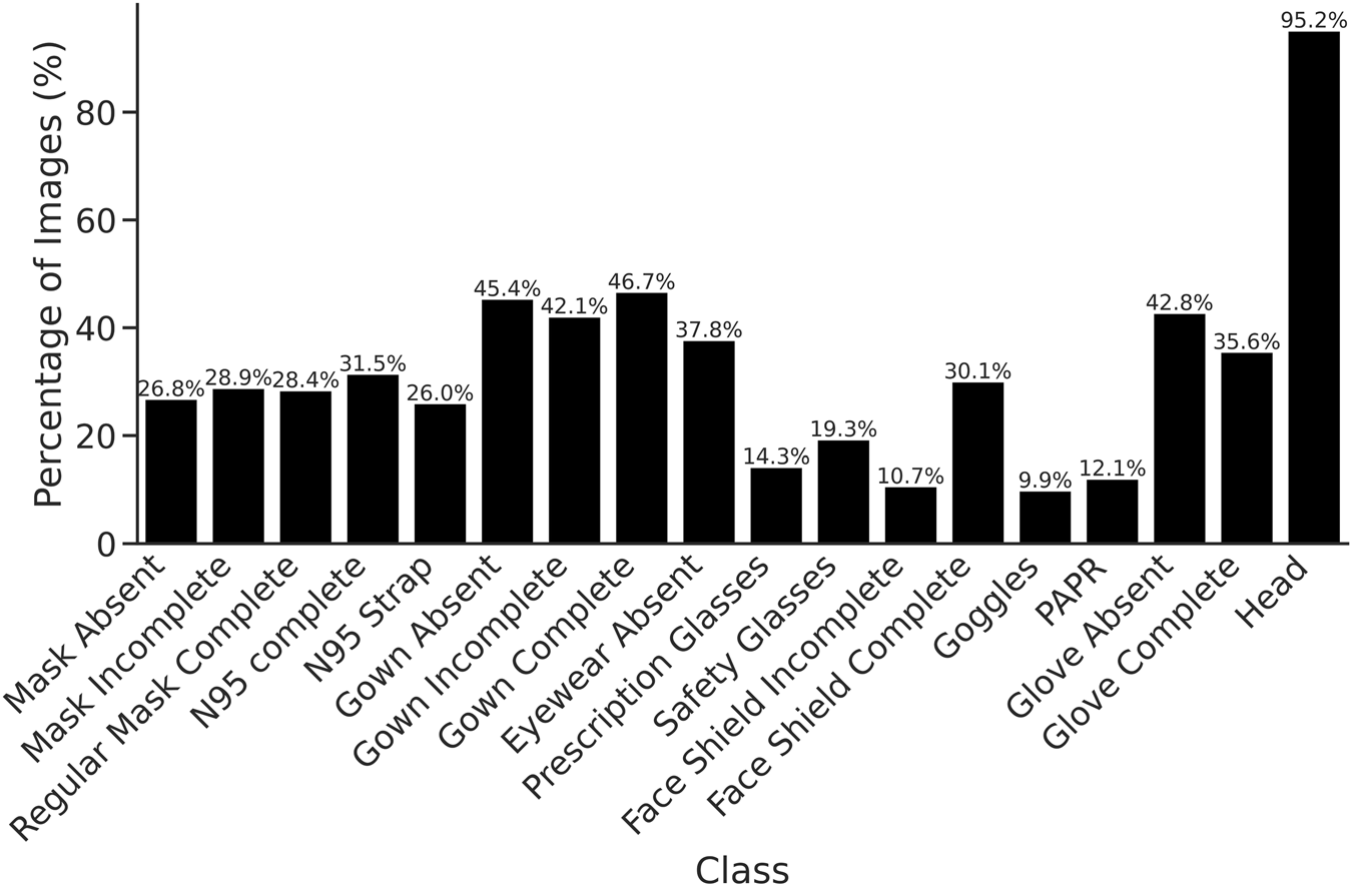
Percentage of images containing each class.

The dataset is available at Zenodo^14^. The extracted annotated frames formed a dataset of 10,034 images and 123,751 object annotations across 17 classes of PPE adherence and nonadherence and one additional class for heads. The annotations were created in Darwin 2.0 (JSON) format. We transformed these data into the Common Objects in Context (COCO) format and stored them in a .json file. The COCO format is a structure preferred for object detection tasks due to its efficacy and common usage^15^ (Table 3). The annotation file consists of three lists: images, annotations, and classes. The images list includes the following attributes for each image: the file name containing the original video name and frame index, image height and width, an isdense flag for scene complexity, and a unique identifier. The annotations list contains 123,751 bounding boxes, each defined by its size and location on the image. The location format includes the x- and y-coordinates of the top-left corner, as well as width and height. Each bounding box was also assigned a PPE adherence or nonadherence class, an image identifier, and a unique identifier. When generating the bounding boxes, we aimed for a representation of PPE categories and instances of adherence and nonadherence to balance each class. Certain classes will have more instances than others because of the variability of adherent and nonadherent PPE usage for different body regions. For example, gown adherence can be only one of three classes, while eye protection can be among seven classes. By applying this approach, we generated combinations of PPE adherence and nonadherence across different PPE categories (Figs. 2, 3). The classes list contains the names and identifiers for the PPE adherence and nonadherence classes and the head class (Table 1). Each class name combines the PPE category and adherence level into an abbreviation (e.g., glove_absent_ha).

**Table 3:** R2PPE JSON file description.

List Name	Length	Attribute	Example
images	10034	file_name	clinical_simulation_5_268.png
		height	1080
		width	1920
		isdense	0
		id	839
annotations	123751	area	5394
		bbox	[466,4,87,62]
		class_id	8
		image_id	942
		id	8709
classes	18	name	Glove_Absent_ha
		id	4

**Fig. 4:**
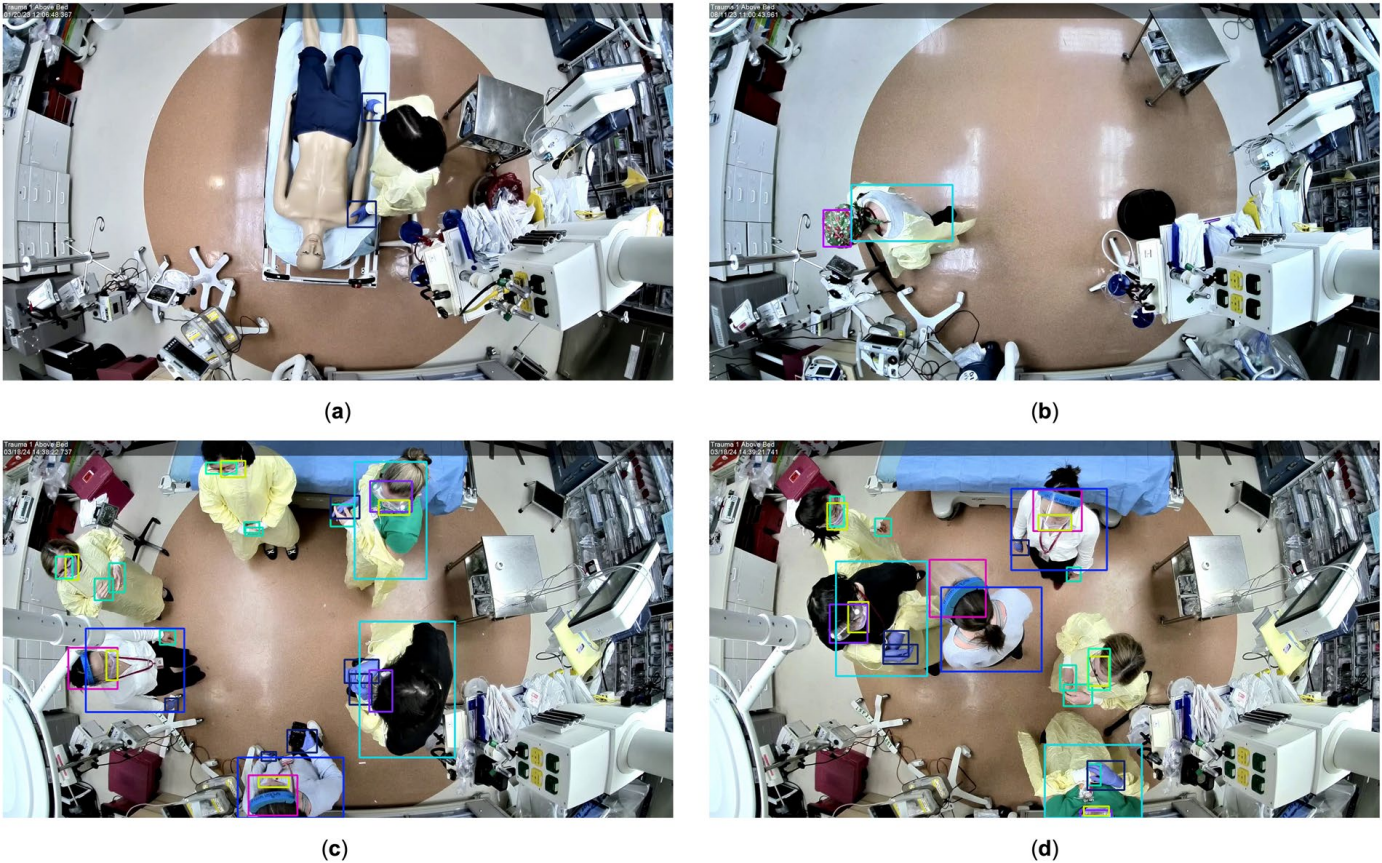
Sample images: (**a**) and (**b**) from R2PPE-L, and (**c**) and (**d**) from R2PPE-H. Images in R2PPE-L contain fewer or less overlapped bounding boxes, while images in R2PPE-H have more densely packed and overlapped bounding boxes.

The R2PPE dataset was categorized into two non-overlapping subsets, R2PPE-L (low-density) and R2PPE-H (high-density), based on data complexity and the density of bounding boxes in images. R2PPE-L contained 6,198 images, showing scenes with an even distribution of bounding boxes for easier detection. R2PPE-H included 3,836 images, featuring densely packed bounding boxes and frequent occlusions, presenting more complex challenges for object detection (Fig. 4). We used the isdense flag in the annotation file to differentiate images from these two subsets: 0 for R2PPE-L and 1 for R2PPE-H. This categorization enables us to systematically analyze and compare the performance of object detection models under varying degrees of scene complexity. The R2PPE dataset at Zenodo^14^ includes three folders: the images folder containing .png files, the annotations folder containing .json files and the videos folder containing the 26 simulation videos.

## Technical Validation

### Bounding Box Analysis

The R2PPE dataset is characterized by a dense concentration of bounding boxes within each image. To show the uniqueness of this density, we compared it with the density of three other datasets: Pattern Analysis, Statistical Modelling and Computational Learning Visual Object Classes (PASCAL VOC)^16^, COCO^15^, and CPPE-5. PASCAL VOC and COCO were selected because these datasets are widely recognized benchmarks in the object detection field. CPPE-5 was selected because it is the most current medical PPE dataset and is most closely aligned with the context of our work. For the R2PPE dataset, the average number of bounding boxes per image is 12.4, which is higher than 2.5 in PASCAL VOC and 7.3 in COCO. R2PPE also features denser annotation compared to CPPE-5, which averages 4.5 bounding boxes per image. The high density in R2PPE results from images with a higher number of adherence and nonadherence classes, along with heavily overlapping bounding boxes.

**Fig. 5:**
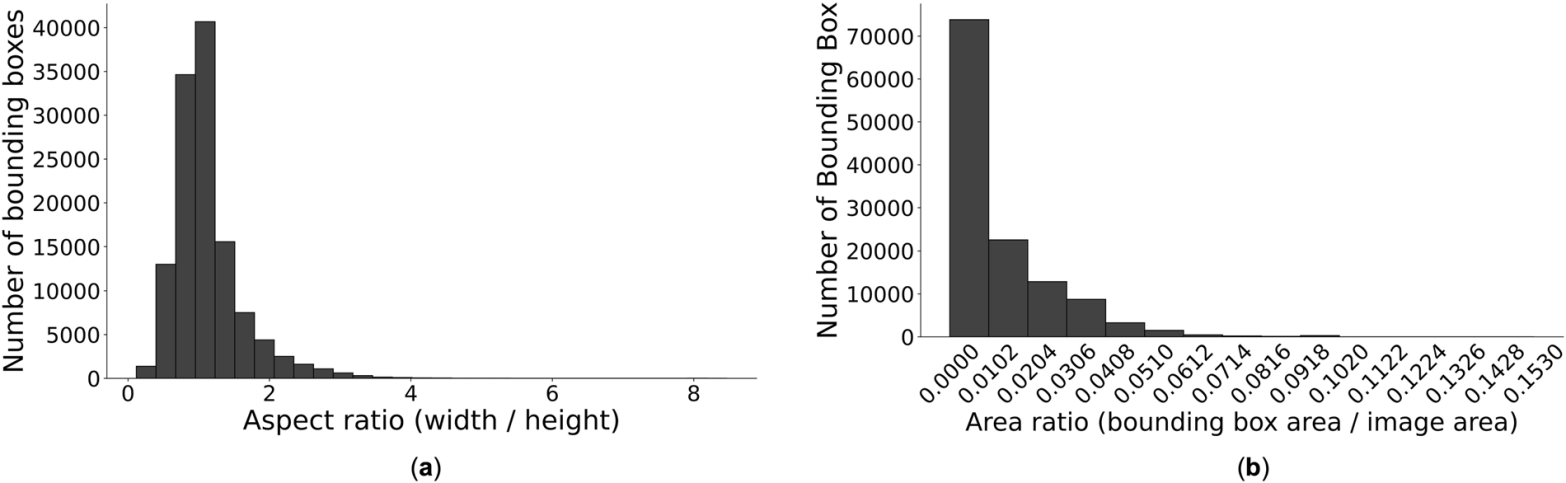
Bounding box aspect and area ratio distributions in the dataset images. (**a**) Bounding box aspect ratio distribution. (**b**) Bounding box area ratio distribution.

Our dataset accounts for the aspect ratio of bounding boxes, calculated by dividing the width of the box by its height. These variations in aspect ratios present challenges for object detection models, especially for those that rely on predefined anchor box shapes and sizes. Detection models may perform less effectively on datasets with a wide range of aspect ratios, while boxes with extreme aspect ratios introduce other detection challenges^17^. For example, boxes with extreme aspect ratios can lead to an imbalanced distribution of feature map resolutions, hindering the model’s ability to learn effective representations required for accurate object detection. A histogram of the aspect ratios derived from the bounding boxes in our dataset shows a predominant concentration around 1, with a tail extending towards higher ratios (Fig. 5a). We also analyzed the ratio of each bounding box’s area to the overall image area (Fig. 5b). Because the frame images are captured by a fixed camera at a high resolution of 1080 × 1920 pixels, the bounding box areas are relatively small compared to the overall image size. Small bounding boxes increase localization errors and reduce the number of features available for classification. For researchers in the object detection field, these small bounding boxes may pose a challenge that requires accurate identification of details within the images.

### Image Density Metrics

We introduce two metrics to highlight the unique characteristics of the R2PPE dataset and to benchmark it against other public datasets. These metrics provide an insight into the challenges in detecting target bounding boxes in images with densely populated annotations. We define local density *D*_*l*_ as a measure of the extent to which each bounding box overlaps with others within the same image, reflecting the concentration of objects in specific image areas. We also introduce global density *D*_*g*_, a measure of the crowdedness of bounding boxes in the entire image. This metric provides a holistic measure of object density across the image space. By measuring density within specific image areas (i.e., locally) and across the entire image space (i.e., globally), we can gain insight into the challenges of achieving accurate object detection in high-density scenarios.

For an image *X*, the function *B*(*X*) yields the number of bounding boxes within it, each designated as *b*_*i*_, with *i* ranging from 1 to *B*. The Intersection over Union (IoU) for any pair of bounding boxes is computed as the ratio of the area of their intersection to the area of their union:

1 $$IoU({b}_{i},{b}_{j})=\frac{I({b}_{i},{b}_{j})}{A({b}_{i})+A({b}_{j})-I({b}_{i},{b}_{j})},$$

where the function *A*(⋅) computes the area of a bounding box, and the function *I*( ⋅, ⋅ ) represents the intersection area between two bounding boxes. We then determine the maximum IoU for a bounding box with any other bounding boxes within the same image. The local density of image *X*, represented as *D*_*l*_, is defined as the average of these maximum IoUs for all bounding boxes present in the image:

2 $${D}_{l}(X)=\frac{1}{N}\mathop{\sum }\limits_{i=1}^{N}\mathop{\max }\limits_{j\in \{1,2,\,...,\,N\}\backslash \{i\}}IoU({b}_{i},{b}_{j}).$$

The global density, *D*_*g*_, is defined as the number of bounding boxes within image *X*:

3 $${D}_{g}(X)=B(X).$$

By integrating the measures of local (i.e., specific areas within an image) and global (i.e., the entire image space) density, we can gain insights into both the number of bounding boxes present in an image and their distribution across the entire image view.

**Fig. 6:**
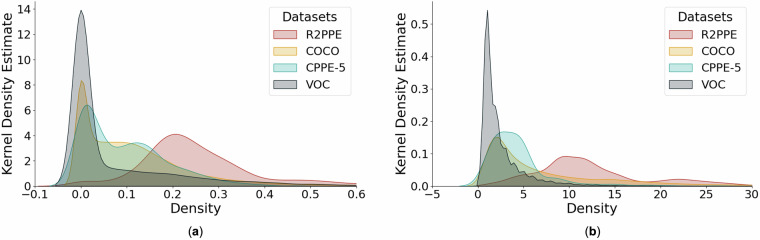
Comparison of R2PPE and three other relevant datasets using local and global density. The x-axis represents density, and the y-axis represents the proportion of data points proximate to the corresponding x-value relative to the total data count. (**a**) Local density *D*_*l*_. (**b**) Global density *D*_*g*_.

To evaluate the distinctions between our dataset and other prevalent public datasets, we determined the local and global densities for images in PASCAL VOC, COCO, CPPE-5 and the R2PPE dataset (Fig. 6). Local density estimations for PASCAL VOC, COCO, and CPPE-5 cluster around zero, indicating that bounding boxes do not overlap in most images (Fig. 6a). In contrast, images in R2PPE mostly have a local density near 0.2. This result implies a higher prevalence of overlapping bounding boxes in the R2PPE dataset. A similar trend is observed for global density (Fig. 6b). PPE datasets, CPPE-5 and R2PPE, exhibit a greater number of bounding boxes per image in comparison to the general-purpose datasets (PASCAL VOC and COCO) because a person wears multiple PPE categories. Our analysis suggests that the R2PPE dataset with its realistic features is more adequate for training object detection algorithms in medical domains than other available datasets.

To encourage targeted algorithm optimization and benchmark the performance of algorithms under different conditions, we divided our dataset into low-complexity (R2PPE-L) and high-complexity (R2PPE-H) segments using local density as a criterion. In real-world scenarios, healthcare workers often either congregate around the bed or move across the room. This difference in provider location and movement creates a complex scene and makes it challenging to draw a clear boundary to separate images with different levels of complexity. To address this challenge, the local density of images can serve as a criterion. We sorted all images in the R2PPE dataset by their local density and categorized them into a simple subset with low density (R2PPE-L) and a difficult subset with high density (R2PPE-H). We selected a threshold of 0.28 based on the local density curve (Fig. 6a). At this threshold, the curve transitions from a steep decline to a more gradual slope. This change in the trend indicates a shift in the density distribution, making 0.28 a natural threshold for dividing the dataset into subsets with distinct density characteristics. Images with local densities surpassing the threshold are classified under R2PPE-H, while others are allocated to R2PPE-L.

### Model Detection Results

The high-density characteristic in the R2PPE dataset poses unique challenges for existing object detection models. To evaluate the adaptability of object detection models to our dataset, we applied several commonly used models. These object detection models are categorized into one-stage^18,19^ and two-stage detectors^20–22^. For our experiments, we selected You Only Look Once (YOLO)^19^ and Faster Region-based Convolutional Neural Network (Faster R-CNN)^20^ as representative baseline models from the one- and two-stage categories, respectively, due to their widespread recognition in the object detection field. We also included the Real-Time Detection Transformer (RT-DETR)^23^ model to represent frameworks based on the emerging use of the transformer architecture^24^. We randomly partitioned the dataset into training and test sets with a ratio of 80:20. Pre-trained on the COCO dataset, the three representative models were finetuned on the R2PPE training set without any modifications to their architectures. We used PyTorch^25^ for all baseline experiments, with the detectron2 library (https://github.com/facebookresearch/detectron2) for Faster R-CNN and the Ultralytics library (https://github.com/ultralytics/ultralytics) for YOLO and RT-DETR. The specific configurations were faster_rcnn_R_101_FPN_3x.yaml for Faster R-CNN, yolov5l.pt for YOLO, and rtdetr-x.pt for RT-DETR. Each model was trained for 80 epochs. We evaluated the performance of each model by calculating the Average Precision (AP) score for each class, specifically employing the AP[0.5:0.05:0.95] metric, a standard evaluation metric derived from the COCO dataset benchmarks. This metric computes the AP by averaging over a range of IoU thresholds, starting from 0.50 to 0.95 in increments of 0.05 (Table 4).

**Table 4:** Performance comparison of baseline models using AP scores for each class in the R2PPE dataset (definitions of PPE class names referred to in Table 1).

	Mask					Gown			Eyewear							Glove		Head	ALL
	MA	MI	RC	NC	S	GA	GI	GC	EA	FC	FI	SG	PG	GG	PR	HA	HC		
Faster R-CNN	22.4	33.0	33.7	33.9	31.7	52.8	55.6	54.6	31.8	44.8	65.5	47.7	41.9	55.8	85.9	42.2	48.9	65.3	47.1
YOLO	27.6	37.1	37.8	38.0	36.2	66.6	68.4	63.2	35.9	52.3	70.0	54.7	47.4	61.6	92.3	51.2	60.2	76.0	54.3
RT-DETR	24.5	35.4	34.0	35.2	35.8	59.4	61.7	60.9	33.1	48.2	68.0	51.7	47.1	57.7	89.2	45.0	51.5	70.7	50.0

In the evaluation of the three object detection models on our dataset, several insights emerged. First, the consistent performance across the three models indicates the integrity of the dataset, showing that a particular detection methodology was not favored. Moreover, across all classes in our dataset, the models achieved AP scores that closely align with their performances on the COCO dataset, with Faster R-CNN at 42%, YOLOv5 at 49% and RT-DETR at 54.8%, despite the COCO dataset containing five times more classes than R2PPE. This similarity in performance and the difference in the number of classes highlight the distinctive challenges our dataset poses. Among the three models, YOLOv5 outperformed its counterparts. This advantage is due to its real-time detection capabilities and direct bounding box predictions, which are particularly effective in handling small and densely packed objects. The feature pyramid network (FPN) in YOLOv5 enhances its ability to detect objects at multiple scales, making it well-suited for high-density, small-object scenarios present in the R2PPE dataset. These results suggest that future work could further explore one-stage detectors for PPE detection in the R2PPE dataset. A common challenge encountered by all models was the detection of mask classes, likely due to smaller bounding box sizes, occlusions and the nuanced differences between “mask absent” and “mask incomplete” classes. This challenge is also an area that deserves special attention in future research.

## Usage Notes

The R2PPE dataset is organized in the COCO format, suitable for common object detection tasks. Tools like PyCocotools (https://pypi.org/project/pycocotools/) and Ultralytics can be used for easy processing of this format. For detailed instructions and further usage information, refer to the code in our GitHub repository.

## Data Availability

All code used to generate and manipulate the dataset, as well as code used in the Technical Validation, is available in a Github repository (https://github.com/yangwanzhao/R2PPE-DATASET).
